# The serum levels of the cytokines involved in the Th17 and Th1 cell commitment are increased in individuals with borderline thrombocytopenia

**DOI:** 10.1186/1756-8722-6-28

**Published:** 2013-04-12

**Authors:** Andreia Maria Camargos Rocha, Cláudia Souza, Gifone Aguiar Rocha, Fabrício Freire de Melo, Nelma Cristina Diogo Clementino, Marília Campos Abreu Marino, Dulciene Maria Magalhães Queiroz

**Affiliations:** 1 Laboratory of Research in Bacteriology, Faculdade de Medicina, Universidade Federal de Minas Gerais, Av. Alfredo Balena, 190, sala 216, Belo Horizonte, 30130-100, Brazil; 2 Hematology Service, University Hospital, Universidade Federal de Minas Gerais, Belo Horizonte, Brazil; 3 Department of Microbiology, Instituto de Ciências Biológicas, Universidade Federal de Minas Gerais, Belo Horizonte, Brazil; 4 Departament of Internal Medicine, Faculdade de Medicina, Universidade Federal de Minas Gerais, Belo Horizonte, Brazil

**Keywords:** Thrombocytopenia, Th1 and Th17 cytokines, *IL2-*330, *IL1RN* VNTR

## Abstract

The definition of immune Thrombocytopenia (ITP) as a peripheral blood platelet count less than 100 × 10^9^/L instead of the historical criteria of 150 × 10^9^/L renders subjects with platelets between 100 and 150 × 10^9^/L without a diagnosis. Here, we demonstrated that these subjects have enhanced levels of proinflammatory cytokines linked to Th1 and Th17 cell response, and are more frequently carriers of polymorphisms in genes that code cytokines involved in the commitment of Th1 and Th17 immune response, when compared with controls, similarly to that observed in patients with ITP.

## To the editor

According to the International Working Group consensus panel [1], primary Immune Thrombocytopenia (ITP) is defined as a peripheral blood platelet count less than 100 × 10^9^/L in the absence of any obvious cause of thrombocytopenia. The recommendation of this value as the threshold for diagnosis of ITP, instead of the historical criteria of 150 × 10^9^/L, was first proposed by Rodeghiero *et al.*[2] based on the results of Stasi *et al.*[3] showing that healthy individuals with incidentally discovered platelet count between 100 and 150 × 10^9^/L have a 10-year probability of developing persistent thrombocytopenia of only 6.9% and of developing autoimmune disorders other than ITP of 12%. However, in addition to the fact that this criterion has not been formally validated yet [4], patients with platelet count between 100 and 150 × 10^9^/L remain without a diagnosis [5]. Recently, our group evaluated the serum cytokine profile of 98 patients with chronic ITP (platelet less than 100 × 10^9^/L) [6] attending the University Hospital, Universidade Federal de Minas Gerais, Brazil and demonstrated higher levels of Th17 cell-related cytokines, in addition to Th1-associated cytokines in the patients than in blood donor controls. We have also demonstrated [7] that *IL1RN* VNTR and *IL2-*330 allele polymorphisms were associated with increased concentrations of IL-1β and of IL-2, respectively and were risk factors for chronic ITP. In this study, we compared serum Th1 and Th17 cytokine profile of 28 individuals (13 female and 15 male, mean age 49.2 years, range from 19 to 79 years) with persistent borderline thrombocytopenia with that of chronic ITP patients and controls we have previously evaluated [6]. None of the included individuals had obvious predisposing conditions or factors associated with thrombocytopenia. The platelet count of the subjects ranged from 101 to 138 × 10^9^/L with a mean (±SD) of 120(±10) × 10^9^/L and the duration of the thrombocytopenia varied from one to 12 years with a mean (±SD) of 4.7(±3.6) years. The serum cytokine concentrations (picogram per milliliter, pg/mL) were assayed in duplicate by ELISA (Biosource, Camarillo, CA). This study was approved by the Ethics Committee of the Institution and informed consent was obtained from all subjects. Data were analyzed with SPSS software package version 17.0 (SPSS Inc., Chicago, IL) and as the data showed significant departures from normality even after log transformation, comparisons among the groups were done by the two-tailed Mann-Whitney U-test. The level of significance was set at p ≤ 0.05.

**Figure 1:**
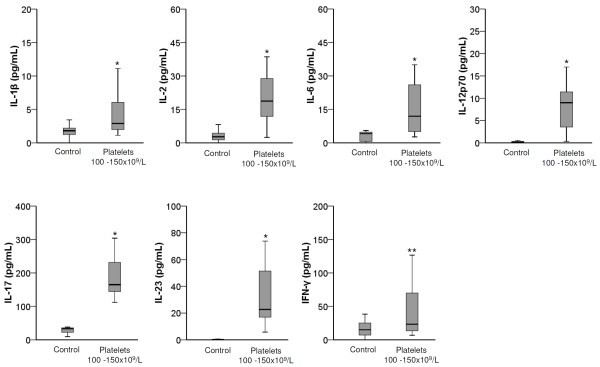
**Box plots representing the serum levels (pg/mL) of IL-1β, IL-2, IL-6, IL-12p70, IL-17, IL-23 and IFN-γ in individuals with platelet count between 100 and 150 × 10^9^/L (n = 28) and controls (n = 150).** The upper and lower limits of the boxes represent the 75^th^ and 25^th^ percentiles, respectively; the horizontal bar across the box indicated the median, and the ends of the vertical lines indicate the minimum and maximum data values * p < 10^-3^; ** p = 0.001.

Of note, the cytokine profile of the studied individuals with platelet count between 100 and 150 × 10^9^/L did not differ from that of ITP patients we have previously studied [6] (IL-17A: 165.0 vs. 169.0 pg/mL, p = 0.55; IL-1β: 2.9 vs. 3.5 pg/mL, p = 0.40; IL-6: 11.9 vs. 14.0 pg/mL, p = 0.32; IL-23: 22.6 vs. 17.7 pg/mL, p = 0.10; IL-2: 18.7 vs. 17.3 pg/mL, p = 0.71; IFN-γ: 23.4 vs. 24.7 pg/mL, p = 0.84; and IL-12p70: 9.0 vs. 7.3 pg/mL, p = 0.46, respectively), but was significantly different from the cytokine profile observed in the healthy blood donors (Figure 1). A 6.3-fold increased serum level of IL-17A, the signature pro-inflammatory cytokine of the Th17 cell, and a 2.5-, 5.0-, and 85.6-fold increased of IL-1β, IL-6 and IL-23, Th17-associated cytokines (p < 10^-3^ for all) were observed in the studied group when compared with the control group (Figure 1). Upregulation of the Th1 cytokines characterized by increased levels of IL-2 (6.3-fold increased, p < 10^-3^), IL-12 p70 (31.0-fold increased, p < 10^-3^) and IFN-γ (2.8-fold increased, p = 0.001) were also observed (Figure 1). Also, the patients with ITP and individuals with platelet count between 100 and 150 × 10^9^/L did not differ in respect to the mean age (p = 0.32), gender (p = 0.20) and duration of thrombocytopenia (p = 0.82). Here we also reanalyzed the data of cytokine gene polymorphisms of 122 patients with platelet count bellow 150 × 10^9^/L published elsewhere [7] by stratifying the group into those with platelet count less than 100 × 10^9^/L and those with platelets between 100 and 150 × 10^9^/L. The presence of at least one allele 2 of *IL1RN* and one G polymorphic allele of *IL2-330* was more frequently observed in the group of subjects with platelet count between 100 and 150 × 10^9^/L (p = 0.04 and p = 0.03, respectively) than in the controls, as well as in the patients with platelets less than 100 × 10^9^/L (p = 0.007 and p = 0.02, respectively) than in the controls, but they did not differ between the patient groups (p = 0.8 and p = 0.7 for *IL1RN* and *IL2* polymorphisms, respectively). The polymorphisms were associated with enhanced concentration of IL-1β (6.3 vs. 2.5 pg/mL, p = 0.002) and IL-2 (22.8 vs. 14.9 pg/mL, p = 0.02) in the IL1RN*2 and *IL2*-330G carriers, respectively, in the group of subjects with platelet count between 100 and 150 × 10^9^/L. Also, in the patients with platelet count less than 100 × 10^9^/L, increased serum concentrations of IL-1β (6.3 vs. 2.8 pg/mL, p < 10^-3^) and IL-2 (22.6 vs. 14.8 pg/mL, p < 10^-2^) were observed in the carriers of IL1RN*2 and *IL2*-330G polymorphic alleles, respectively. In conclusion, individuals with platelet count between 100 and 150 × 10^9^/L have enhanced levels of proinflammatory cytokines linked to Th1 and Th17 cell response, and are more frequently carriers of polymorphisms in genes that encode cytokines involved in the commitment of Th1 and Th17 immune response, similarly to that observed in patients with chronic ITP, which points to the need of a careful follow-up of this group of individuals and to search for pathogenic mechanisms associated with this condition.

## Competing interests

The authors declare that they have no competing interests.

## Authors’ contributions

AMCR and DMMQ were the principal investigators and take primary responsibility for this paper; AMCR and DMMQ designed the study, done the statistical analysis and wrote the paper. CS, NCDC and MCAM recruited the patients. GAR helped to write the paper; FFM performed the laboratory work. All authors read and approved the final manuscript.

## References

[B1] Provan D Stasi R Newland AC Blanchette VS Bolton-Maggs P Bussel JB Chong BH Cines DB Gernsheimer TB Godeau B Grainger J Greer I Hunt BJ Imbach PA Lyons G McMillan R Rodeghiero F Sanz MA Tarantino M Watson S Young J Kuter D International consensus report on the investigation and management of primary immune thrombocytopenia Blood 2010 115 168 186 10.1182/blood-2009-06-225565 19846889

[B2] Rodeghiero F Stasi R Gernsheimer T Michel M Provan D Arnold DM Bussel JB Cines DB Chong BH Cooper N Godeau B Lechner K Mazzucconi MG McMillan R Sanz MA Imbach P Blanchette V Kühne T Ruggeri M George JN Standardization of terminology, definitions and outcome criteria in immune thrombocytopenic purpura of adults and children: report form an international working group Blood 2009 113 2386 2393 10.1182/blood-2008-07-162503 19005182

[B3] Stasi R Amadori S Osborn J Newland AC Provan D Long-term outcome of otherwise healthy individuals with incidentally discovered borderline thrombocytopenia Plos Medicine 2006 3 388 394 10.1371/journal.pmed.0030024 PMC1326262 16401142

[B4] Neunert C Lim W Crowther M Cohen A Solberg L Jr Crowther MA The American Society of Hematology 2011 evidence-based practice guideline for immune thrombocytopenia Blood 2011 117 4190 4207 10.1182/blood-2010-08-302984 21325604

[B5] Grace RF Long M Kalish LA Neufeld EJ Applicability of 2009 international consensus terminology and criteria for immune thrombocytopenia to a clinical pediatric population Pediatr Blood Cancer 2012 58 216 220 10.1002/pbc.23112 21674757 PMC3175326

[B6] Rocha AMC Souza C Rocha GA Melo FF Clementino NCD Marino MCA Bozzi A Silva ML Martins-Filho OA Queiroz DMM The levels of IL-17A and of the cytokines involved in the Th17 cell commitment are increased in patients with chronic Immune Thrombocytopenia Haematologica 2011 96 1560 1564 10.3324/haematol.2011.046417 21972211 PMC3186321

[B7] Rocha AMC Souza C Rocha GA Melo FF Saraiva ISB Clementino NCD Marino MCA Queiroz DMM *IL1RN* VNTR and *IL2*-330 polymorphic genes are independently associated with chronic immune thrombocytopenia Br J Haematol 2010 150 679 684 10.1111/j.1365-2141.2010.08318.x 20626741

